# New and Emerging Biomarkers in Chronic Kidney Disease

**DOI:** 10.3390/biomedicines13061423

**Published:** 2025-06-10

**Authors:** Mikołaj Dopierała, Nadja Nitz, Oliwia Król, Karolina Wasicka-Przewoźna, Krzysztof Schwermer, Krzysztof Pawlaczyk

**Affiliations:** Department of Nephrology, Transplantology and Internal Medicine, Poznan University of Medical Sciences, 61-701 Poznan, Poland; nadelek01@gmail.com (N.N.); oliwia.krol@usk.poznan.pl (O.K.); k.wasicka@gmail.com (K.W.-P.); kpawlac@ump.edu.pl (K.P.)

**Keywords:** inflammation, kidney biomarkers, chronic kidney disease, cardiorenal syndrome risk, kidney physiopathology, kidney injury

## Abstract

Chronic kidney disease (CKD) represents a major and widespread global health challenge. It affects over 800 million people worldwide, which is approximately 13% of the world’s population. Over the past 20 years, it has consistently ranked among the leading causes of death. As a result of its typically painless and asymptomatic presentation in the early stages of the disease, CKD is frequently diagnosed late, when the patient is already suffering from serious complications. In recent years, studies have identified novel biomarkers associated with the pathophysiology of CKD, including chronic inflammation, tubular injury, and CKD-related outcomes such as bone and mineral metabolism disorders, cardiovascular events, and all-cause mortality. Identifying and using these emerging biomarkers—like kidney injury molecule, N-acetyl–D-glucosaminidase, ficolins, the NLRP3 (nucleotide-binding domain, leucine-rich–containing family, pyrin domain–containing-3) inflammasome, soluble suppression of tumorigenicity-2, galectin-3, growth differentiation factor-15, soluble urokinase-type plasminogen activator receptor, sclerostin, the Dick-kopf proteins, and indexes such as the systemic inflammation response index—may lead to a significant advancement in early diagnosis, risk stratification, and personalized treatment strategies for CKD patients. Despite their potential, the routine clinical use of these novel biomarkers remains limited due to challenges such as high costs and the lack of standardized testing methods. There is still considerable room for advancement in both the diagnosis and management of CKD. Hopefully, increasingly more new biomarkers will become usable in clinical practice, ultimately improving care quality and outcomes for patients with CKD.

## 1. Introduction

Chronic kidney disease (CKD) is a widespread global health issue affecting approximately 13% of the world’s population, which means that it afflicts over 800 million people [1]. It has remained one of the most prominent causes of death over the past two decades [2,3]. The rise in risk factors, particularly diabetes mellitus, hypertension, cardiovascular disease, and obesity, compounds the burden of CKD in the population [1]. CKD is defined as abnormalities of kidney structure or function, present for a minimum of 3 months, with implications for health [4]. The pathophysiology of CKD is complex, but the main pathological hallmarks include fibrosis, tubular atrophy, and chronic inflammation. The progression of this process is primarily driven by proteinuria, hypoxia, and oxidative stress [5]. Early detection of CKD provides opportunities to effectively attenuate the progression of renal dysfunction and prevent outcomes associated with CKD. However, primarily due to its typically painless and initially asymptomatic presentation, CKD is frequently diagnosed in the later stages. Currently, in clinical practice, the diagnosis and staging of CKD are made by estimating glomerular filtration rate (GFR) using creatinine and cystatin C or by albuminuria (ACR) [4]. These diagnostic criteria reflect renal function based on glomerular filtration. However, studies from recent decades identified novel biomarkers associated with the pathophysiology of CKD, including chronic inflammation, tubular injury, and CKD-related outcomes. These markers enable a more specific representation of overall kidney function and CKD complications.

## 2. Methodology

In this narrative review, we attempted to provide a comprehensive overview of recent advances in the identification of novel biomarkers for CKD. We conducted a comprehensive search of the relevant literature, mainly studies published in the last decade. Both clinical studies (observational studies, cohort studies, clinical trials) and preclinical research (animal models, mechanistic studies) were included to provide a broad understanding of biomarker relevance from basic science to clinical application. Studies not available in English, conference abstracts without full texts, and data still awaiting publication were excluded. 

We strived to include a wide array of biomarkers and present an integrated view on both established and newly emerging biomarkers, with special attention to those that are rarely mentioned in previous reviews, such as ficolins or Dick-kopf proteins, and others that have only recently gained attention in CKD research, like inflammatory indices. Through a comprehensible pathophysiological framework and extending the understanding of biomarkers’ functions beyond their primary roles, we tried to place a focal point on the translation of these into clinical practice. 

A major limitation of this review is the fact that, given our focus on covering a broad spectrum of biomarkers, the depth of discussion for each individual marker was necessarily limited.

## 3. Biomarkers of Tubular Secretion

Creatinine, cystatin C, and estimated glomerular filtration rate (eGFR) are the primary parameters used for the definition and staging of chronic kidney disease (CKD). These markers reflect renal function based on glomerular filtration. However, their utility is limited, particularly in detecting the early stages of CKD [6]. Consequently, there is an urgent need to identify more sensitive biomarkers for the early detection of CKD that may more comprehensively represent overall kidney function. Considering tubular injury as a key mechanism in the progression of CKD [7], biomarkers of tubular secretion may prove useful for diagnosing this condition.

Kidney injury molecule-1 (KIM-1) is a transmembrane glycoprotein with undetectable expression in the normal kidney [8]. KIM-1 consists of an immunoglobulin-like domain and an extracellular mucin domain. Renal injury factors lead to the upregulation of KIM-1 expression by proximal tubule epithelial cells. The mucin domain is shed, resulting in elevated levels of KIM-1 in urine and plasma [9,10]. KIM-1 serves as a sensitive biomarker of proximal tubule injury in both acute kidney injury (AKI) and chronic kidney disease (CKD) [10,11,12]. In a cross-sectional study by Waikar et al. (2016), a strong positive correlation was demonstrated between urinary KIM-1 level and the albumin-to-creatinine ratio (ACR) in individuals with and at risk for CKD (95% CI: 0.15-0.17), suggesting its potential role in the diagnosis and prognosis of CKD [13]. Urinary KIM-1 levels increased progressively with declining eGFR, until eGFR fell below 15 mL/min/1.73 m², likely reflecting a reduction in tubular mass responsible for KIM-1 expression [13]. Waikar et al. also reported reduced urinary KIM-1 levels in users of angiotensin-converting enzyme inhibitors (ACEIs) or angiotensin II receptor blockers (ARBs), which correlates with the nephroprotective properties of these classes of medications [13,14,15]. The upregulation of KIM-1 has also been observed in various glomerulopathies, including diabetic glomerulopathy and IgA nephropathy [16,17]. Chronic KIM-1 expression is a component of the pathogenesis of chronic kidney disease. It plays a significant role in the progression of inflammation and tubulointerstitial fibrosis in renal tissue, partly by promoting the secretion of monocyte chemoattractant protein-1 (MCP-1) [18]. In conclusion, KIM-1 is not only a sensitive biomarker for CKD but can also be a potential therapeutic target in kidney disease [18,19].

N-acetyl–D-glucosaminidase (NAG) represents another urinary biomarker of proximal tubular injury [20]. This lysosomal enzyme has been investigated as a potential predictor of diabetic nephropathy, highlighting the role of tubular injury in the early stages of diabetic kidney disease [21,22]. Urinary NAG excretion can be detected before the appearance of microalbuminuria and macroalbuminuria in diabetic kidney disease (DKD) [23]. NAG is not filtered through the glomerulus due to its relatively high molecular weight, but it may appear in the urine as a consequence of tubular injury, including exposure to high urinary glucose [24,25]. Urinary NAG levels are not only significantly higher in diabetic patients but also reflect renal impairment, indicating the promising utility of NAG in monitoring diabetic kidney disease (DKD) [26]. Furthermore, Kim et al. demonstrated that urinary NAG may reflect glycemic control in patients with type 2 diabetes mellitus due to its correlation with glucometabolic parameters [27]. More recently, the application of NAG in the real-time monitoring of kidney function in DKD was presented. Ten et al. fabricated a NAG-responsive NIR-II fluorescent nanoprobe that specifically interacts with NAG to produce NIR-II fluorescence signals, enabling the noninvasive detection of kidney dysfunction [28].

Uromodulin, also known as Tamm–Horsfall protein, is a renal-specific protein expressed exclusively by the thick ascending limb of the loop of Henle and the distal convoluted tubule [29]. It is a major protein present in the urine of healthy individuals [30,31]. Numerous studies demonstrated multiple physiological functions of uromodulin, including ion reabsorption in the thick ascending limb, blood pressure control, protection against kidney stone formation, and immunomodulation [32,33,34,35,36,37]. Additionally, uromodulin prevents bacterial urinary tract infections. It counteracts the adherence of Escherichia coli to the urothelial surface by binding specifically to type 1 fimbriated Escherichia coli [38,39].

Both urinary and serum uromodulin have been investigated in the context of CKD. Distal tubular damage leads to lessened urinary excretion of Tamm–Horsfall protein [40,41,42]. It is also modulated by common variants in the UMOD gene, which encodes uromodulin [43]. In a case–control study, Köttgen et al. identified an association between a single-nucleotide polymorphism in the UMOD region (rs4293393) and elevated uromodulin concentrations as a predictor of CKD onset [44]. Urinary uromodulin serves as a biomarker of kidney tubulointerstitial damage [45], a key pathological mechanism affecting CKD development [46]. Urinary uromodulin level has been shown to predict rapid progression to end-stage kidney disease (ESKD) in CKD patients within 1 year [47].

According to research, serum uromodulin may also serve as a biomarker for kidney function assessment [48,49,50]. In an observational study including 170 patients with CKD, Fedak et al. demonstrated a strongly positive correlation between serum uromodulin concentrations and eGFR [51]. Importantly, the relationship between serum uromodulin and eGFR was independent of age, sex, BMI, and body surface area, enhancing the potential diagnostic utility of this protein [51]. Serum uromodulin, as a marker of tubular secretion, represents functional nephron mass and kidney function as a whole [49,52]. In this way, serum uromodulin can help identify the early stages of CKD, including the creatine-blind range of CKD [52,53]. Similarly to urinary uromodulin, serum uromodulin may be used to identify high-risk groups for the incidence of ESKD in CKD patients [54]. Its level is also associated with a common CKD outcome–-cardiovascular disease [55].

What is more, uromodulin has been implicated in various health conditions associated with CKD. Low serum uromodulin levels may be correlated with kidney function and renal disease activity in lupus nephritis and ANCA-associated glomerulonephritis [56,57]. In the course of Fabry disease, uromodulin excretion is also disturbed, ranging from normal to markedly decreased or even absent. However, a study of 15 male Fabry disease patients has shown that enzyme replacement therapy leads to the normalization of urinary uromodulin excretion [58]. Mutations in the gene encoding uromodulin underlie medullary cystic kidney disease type 2 and familial juvenile hyperuricemic nephropathy [59]. In these kidney diseases, impaired uromodulin export dynamics lead to an intracellular accumulation of this protein in the tubular epithelium of the thick ascending limb of Henle’s loop. Consistently, urinary uromodulin excretion is reduced [60].

In conclusion, uromodulin is a promising biomarker for CKD, including the early stage of CKD. Furthermore, given its important role in maintaining kidney function, this protein may serve as a predictor of CKD onset and progression and could represent a novel target for therapeutic intervention.

The Klotho protein, another potential biomarker for CKD, was first identified in 1997 as an anti-aging molecule [61]. Subsequent research has established the role of Klotho deficiency in the aging process and the pathogenesis of age-related diseases [62,63,64,65]. This protein is predominantly expressed in the kidney, specifically in both the proximal and distal tubules [66]. Additionally, it has also been detected in the epithelium of the choroid plexus in the brain, as well as in the heart, pituitary gland, parathyroid gland, and other tissues [67]. Klotho is a transmembrane protein that functions as a coreceptor for fibroblast growth factor (FGF) [68]. Its extracellular domain undergoes shedding, resulting in the release of a soluble form into the bloodstream and urine [69]. Many studies have investigated the correlation between circulating soluble Klotho levels and kidney function. The majority indicated a positive correlation between serum and urine Klotho levels and estimated glomerular filtration rate (eGFR) in CKD [70,71,72]. In contrast, Seiler et al. reported no link between plasma Klotho and kidney function [73]. These divergences are likely due to variability in assay methods and the heterogeneity of CKD populations. However, Klotho is a substantiated renoprotective molecule, and its deficiency plays an important role in CKD progression [74,75,76]. It may serve as a potential biomarker for the early stages of CKD and a therapeutic target, and it can also predict CKD complications, though there is a requirement for the standardization of measurement methods for its clinical application.

Proteins in the Dickkopf (DKK) family are antagonists and modulators of the Wnt/beta-catenin signaling pathway [77]. This family encompasses four key proteins: Dick-kopf-1, Dick-kopf-2, Dick-kopf-3, and Dick-kopf-4 [77]. Of these, Dick-kopf-1 and Dick-kopf-3 have recently emerged as potentially significant novel biomarkers in the course of not only the diagnosis and progression of CKD complications due to their direct mechanism of action, as well as their involvement in "cross-talk" with other body tissues, but also as a potential prognostic marker [78,79].

Dickkopf-3 (DKK-3) is a urinary glycoprotein secreted as a response to stress and is expressed in tubular cells after injury [80], serving as a marker for ongoing tubular stress with the potential of serving as a biomarker of CKD progression; currently, several studies have confirmed its potential as an ongoing biomarker of renal injury [81]. Urinary levels of DKK-3 (uDKK-3) have been associated with poor renal survival in CKD and estimated eGFR decline [82]. High urinary levels of DKK-3 have demonstrated increased eGFR loss [83] in resistant hypertension CKD patients, where eGFR loss was higher even up to 24 months later [82]. This was especially the case when uDDK-3 was greater than or equal to 400 pg/mL, resulting in a statistically significant eGFR decline as well as the more frequent appearance of proteinuria [82]. uDDK-3 has also been correlated with an increased risk of CKD progression in patients diagnosed with chronic obstructive pulmonary disease (COPD) [83].

Dickkopf-1, as a protein involved in the regulation of bone metabolism, will be further discussed in subsequent sections of this review.

The biomarkers mentioned in the text are presented in Table 1.

## 4. Inflammatory Biomarkers

Impaired kidney function leads to the accumulation of uremic toxins and oxidative stress, activating pro-inflammatory pathways. Over the years, many inflammatory markers have been associated with chronic kidney disease. They are not only indicators of systemic inflammation but can also be used as predictors of cardiovascular disease, stroke, progression of kidney damage, and loss of renal function, as well as overall mortality [84,85,86,87,88,89,90,91,92]. Elevated levels of biomarkers such as C-reactive protein (CRP) [93,94,95,96,97,98,99,100,101,102,103], tumor necrosis factor alpha (TNF-a) [93,94,104,105,106,107,108,109,110,111,112,113,114], interleukin-6 (IL-6) [93,99,101,113,115,116,117,118,119,120], interleukin-1b (IL-1b) [102,103,113,121,122], interleukin-10 (IL-10) [93,123,124,125], fibrinogen [103,114,115,126,127], serum amyloid A (SAA) [128,129,130,131], and Transforming Growth Factor-Beta (TGF-β) [132,133] are well known to be correlated with CKD.

On the other hand, a high concentration of some inflammatory markers may seem to play a therapeutic role. For example, interleukin-10 (IL-10) may reduce fibrosis and inflammation [123,124].

In recent years, new inflammatory molecules and indexes have emerged as biomarkers for CKD. Below are some key findings regarding their potential role in CKD.

Ficolins are pattern recognition molecules. They play a role in pathogen recognition, complement activation, and inflammation regulation. There are three main types of ficolins: M-ficolin (ficolin-1), L-ficolin (ficolin-2) and H-ficolin (ficolin-3). Mainly, L-ficolin and H-ficolin were studied in relation to CKD. The pattern recognition molecules within the complement system, such as H-ficolin and mannan-binding lectin (MBL), recognize specific molecular patterns on the surface of microorganisms and, by binding them, can activate the lectin pathway of the complement system. This, in turn, leads to inflammation. Hyperglycemia alters glycans enzymatically and non-enzymatically by the formation of advanced glycation end-products. These glycan alterations may be the cause of a harmful complement auto-attack initiated by pattern recognition molecules. Østergaard et al., in their article, suggested that higher levels of H-ficolin were associated with diabetic kidney disease progression. However, while the association with DKD progression was independent of other well-established risk factors, the effect disappeared after adjusting for triglyceride levels. Furthermore, in the studied cohort, high concentrations of H-ficolin predicted diabetes-related mortality but were not connected to all-cause mortality and cardiovascular events [134]. Elevated H-ficolin levels at the time of kidney transplantation were found to be an independent and significant risk factor for shorter graft survival [135]. What is more, L-ficolin gene polymorphism was shown to influence kidney allograft functions [136].

The NLRP3 (nucleotide-binding domain, leucine-rich–containing family, pyrin domain–containing-3) inflammasome is a multiprotein complex and a component of the innate immune system. By triggering the release of pro-inflammatory cytokines such as IL-1B and IL-18, it plays a role in inflammation, fibrosis, and the progression of CKD. Research has shown that through damaged mitochondria and an elevated production of reactive oxygen species (ROS), the NLRP3 inflammasome is activated in uremic patients treated with dialysis [137]. Other authors have suggested that NLRP3 inflammasome inhibition may be a promising therapeutic target in hyperoxaluria and nephrocalcinosis [121], as well as reducing renal fibrosis [138].

Serum tumor necrosis factor receptor 1 and 2 (TNFR1 and TNFR2) levels are related to eGFR in healthy subjects, which highlights their potential as early biomarkers for CKD [139]. In a study by Oh et al., elevated concentrations of tumor necrosis factor receptors (TNFRs) were correlated with an increased risk of progression in immoglobulin A nephropathy (IgAN). Circulating TNRFs can be early biomarkers to predict the severity and clinical outcome of IgAN [140]. TNFRs can also be a prognostic biomarker for diabetic kidney disease [107]. Further studies focused on TNFRs evaluated Black individuals with hypertension-attributed CKD and older adults with type 2 diabetes and albuminuria, proving that increases in TNFR1 and TNFR2 were associated with worsening kidney function in those groups [141].

Calprotectin is circulation damage-associated molecular pattern protein, considered an acute-phase protein. It originates mainly from myeloid cells and is released during neutrophil degranulation. Higher levels of circulating calprotectin are linked to increased risk of new-onset CKD [142]. Plasma calprotectin was also identified as a contributor to vascular calcification, cardiovascular (CV) outcomes, and mortality in patients with CKD [143,144].

The systemic inflammation response index (SIRI) is a novel predictive biomarker derived from the ratio of peripheral neutrophil, monocyte, and lymphocyte counts, calculated as (neutrophil count × monocyte count/lymphocyte count). The SIRI, as well as other indexes like the neutrophil-to-lymphocyte ratio (NLR), the platelet-to-lymphocyte ratio (PLR), and the systemic immune-inflammation index (SII), were found to be significantly increased in CKD stage 5 compared to other CKD stages [145,146]. A high SIRI is linked to a greater risk of CKD in patients with hypertension. As the SIRI increases, the risk of CKD in hypertensive patients also rises [147]. Furthermore, an elevated SIRI is linked to an increased risk of both all-cause and cardiovascular mortality in patients with CKD. Regular monitoring of the SIRI can aid in the early identification of high-risk patients, enabling prompt and targeted interventions [148]. Some studies have shown that it can be especially significant in the early stages (I-III) of CKD [87].

An elevated NLR is linked to increased proteinuria, higher serum creatinine levels, reduced eGFR, and a greater prevalence of advanced CKD. Therefore, a high NLR indicates a more advanced stage of CKD and may serve as a biomarker for predicting its progression [149]. NLR can also be used as a complementary prognostic marker for cardiovascular risk evaluation in patients with moderate-to-severe chronic kidney disease [150] and a predictor of cardiovascular and all-cause mortality in hemodialysis patients [151].

Hypoalbuminemia, a well-known sign of protein-energy wasting (PEW) or malnutrition, is frequently seen in individuals with CKD and is linked to an increased risk of cardiovascular complications. Recently, the C-reactive protein to albumin ratio (CAR) has emerged as a novel marker for PEW, as this condition appears to stem not only from insufficient dietary intake but also from persistent systemic inflammation. Measurement of CAR may be useful in clinical practice because this newly introduced surrogate marker of the PEW is simple and can be easily obtained in routine practice. In their research, Takahashi et al. showed that the pre-procedural CRP/albumin ratio could predict both the risk of amputation and mortality after lower extremity revascularization and could stratify the risk in HD patients with PAD [152]. In other studies, a higher CAR was found to be an independent risk factor for diabetic nephropathy [153] and CKD in general [154]. CAR has the potential to become a key biomarker for offering new avenues for advancing early risk stratification, precision therapies, personalized management, and preventive strategies in CKD [155].

Although increased levels of inflammatory indices such as the SIRI or NLR correlate with the progression of CKD, as well as with worsening outcomes in CKD patients, it is important to note that this association does not always imply a direct causal relationship. Systemic inflammation may be a consequence of underlying conditions such as malignancies, infections, or autoimmune diseases, which also contribute to CKD progression. In those cases, the inflammation may not be primarily caused by CKD or be the main reason for CKD progression.

The biomarkers mentioned in the text are presented in Table 2.

## 5. Cardiovascular Biomarkers

CKD is a common condition associated with significant cardiovascular morbidity and mortality. Cardiovascular disease (CVD) is a leading cause of death in CKD, accounting for 40% to 50% of all deaths among individuals with advanced and end-stage kidney disease [156]. Novel cardiovascular biomarkers may aid in the early detection of cardiovascular complications and the prediction of CKD progression.

Troponin is a component of cardiac muscle thin filaments and consists of three isoforms: Troponin T (TnT), Troponin I (TnI), and Troponin C (TnC). These isoforms are associated with cardiac injury and various cardiovascular diseases, such as stroke, pulmonary embolism, acute perimyocarditis, and arrhythmias. Circulating troponin levels are widely used in diagnosing these conditions, particularly acute coronary syndromes. [157] Several studies have reported that elevated levels of cardiac troponin T (cTnT) and I (cTnI) are frequently observed in CKD patients, not related to the presence of acute coronary syndromes [158,159]. Furthermore, increased high-sensitivity troponin T (hsTnT) is associated with a higher risk for CKD progression and greater cardiovascular mortality in CKD participants [160,161].

N-terminal pro-B-type natriuretic peptide (NT-proBNP) is a well-established biomarker of heart failure used for diagnosis, risk assessment, and monitoring of disease progression. Studies have demonstrated that higher NT-proBNP levels strongly predict heart failure, and decreased NT-proBNP levels are associated with a lower risk of mortality in CKD populations [160,162]. A recent study has shown a negative correlation between NT-proBNP levels and kidney function in CKD patients. Furthermore, the same study revealed an association between NT-proBNP and an increased risk of kidney disease progression in CKD patients at stages 1–3 without heart failure (HF) [163]. The latest research uncovered that the same NT-proBNP concentration in patients with heart failure and impaired kidney function predicts a higher absolute risk of complications compared to those with normal kidney function [164].

Galectin-3 is a β-galactoside-binding lectin implicated in various biological processes involved in inflammation, fibrosis, cardiac dysfunction, and remodeling. Elevated levels of galectin-3 have been associated with an increased risk of heart failure and its morbidity. Furthermore, several studies suggest that galectin-3 plays a potential role in identifying patients at high risk of cardiac dysfunction before clinical symptoms manifest [165,166,167]. In renal disease, elevated serum galectin-3 concentrations are associated with the onset of CKD, development of renal fibrosis, and rapid renal function decline [168]. According to Kim et al., serum galectin-3 levels showed a direct correlation with both serum creatinine (Cr) levels and the urine protein-to-Cr ratio in CKD patients [169]. Recent studies reveal the potential of galectin-3 as a biomarker for kidney disease progression and its implications in cardiovascular morbidity and mortality among CKD patients [170,171].

Soluble suppression of tumorigenicity-2 (sST-2) is a soluble isoform of ST2, an interleukin-1 receptor-like 1 and member of the interleukin-1 receptor family. Interaction between interleukin -33 (IL-33), the transmembrane ST2 receptor (ST2L), and sST-2 plays a significant role in the inflammatory response to cardiac stress, and sST-2 is strongly associated with myocardial fibrosis, remodeling, and heart failure progression [172,173]. Several studies have reported that elevated serum levels of sST-2 are correlated with progression and higher risk of mortality in CKD participants [169,174] and increased risk of cardiovascular mortality in dialysis patients. Furthermore, sST-2 levels are not correlated with eGFR in CKD patients, indicating that sST2 is the least impacted by declining kidney function. These findings suggest its potential as a reliable biomarker for patients with cardiovascular disease and coexisting chronic kidney disease [175].

Recent reports reveal that elevated concentrations of sST2 and Gal-3 are associated with an increased cardiothoracic ratio (CTR) in CKD patients. This relationship may enable better cardiovascular risk evaluation for CKD patients [176].

Growth differentiation factor-15 (GDF-15) is a stress-responsive cytokine elevated in conditions associated with inflammation and oxidative stress, as well as in different types of cardiovascular events like heart failure and atrial fibrillation. Recently, GDF-15 has been described as a promising biomarker of atherosclerosis [177]. In terms of kidney disease, available studies indicate that GDF-15 is involved in the pathophysiology of CKD, diabetic nephropathy, and kidney cancer [178]. Furthermore, elevated levels of GDF-15 are associated with adverse renal outcomes and may serve as a potential biomarker for predicting the progression of chronic kidney disease (CKD) and its cardiovascular complications [179,180].

Soluble urokinase-type plasminogen activator receptor (suPAR) is a soluble form of urokinase-type plasminogen activator receptor (uPAR) involved in systemic inflammation and immune activation [181]. suPAR is a membrane bound receptor that interacts with and activates integrins in diverse cell types, such as podocytes, tubular epithelial cells (TECs), and fibroblasts, leading to a loss of glomerular filtration barrier integrity and podocyte damage.

Elevated suPAR levels have been associated with an increased risk of cardiovascular disease and kidney function decline in CKD patients [182,183]. Recent studies suggest that higher suPAR levels correlate with greater mortality and cardiovascular events, making suPAR a significant predictor for improving risk stratification and early intervention in CKD populations.

The combination of NT-proBNP, cardiac troponins, galectin-3, sST-2, GDF-15, and suPAR represents a promising approach for a more comprehensive cardiovascular risk assessment and mortality prediction in CKD patients. These biomarkers also demonstrate the potential of predicting CKD progression and guiding future therapeutic strategies.

The biomarkers mentioned in the text are presented in Table 3.

## 6. Bone and Mineral Metabolism Biomarkers

Chronic kidney disease (CKD) is associated with a variety of complications either secondary to its progression or as a consequence of the worsening renal function characterizing the disorder. Amongst these, cardiovascular diseases (CVDs), endocrine disorders, metabolic imbalances, mineral and bone disorders (CKD-MBD), and cognitive impairments have been identified [184]. All of the aforementioned have their own established biomarkers used for diagnosis and disease monitoring. However, recent research suggests that these biomarkers may also be used to predict CKD progression and improve prognostic accuracy [185].

Traditional biomarkers for CKD-MBD have included parathyroid hormone (PTH), calcium, alkaline phosphatase, cholecalciferol, and vitamin D [186]. Yet, emerging evidence suggests the presence of novel biomarkers that may provide insights into disease progression and these associated complications [185]. This includes Fibroblast Growth Factor 23 (FGF-23), Sclerostin, Dickkopf-1, and Dickkopf-3, which have emerged as promising biomarkers for CKD-MBD.

PTH, the parathyroid hormone, an 84-amino-acid single-chain peptide, plays a key role in bone metabolism by promoting calcium reabsorption in the kidneys, stimulating osteoclast activity in the bones, as well as enhancing intestinal calcium absorption through the activation of calcitriol.

The parathyroid hormone can be assessed in several forms, including whole PTH, intact PTH, and via the whole/intact PTH ratio [187]. In CKD, the impairment of renal function disrupts its mechanism, leading to secondary hyperparathyroidism (SHPT) [188]. Due to declining renal function and the subsequent decline in GFR, phosphate accumulates, and this stimulates the release of PTH and binds free calcium in the blood reducing its levels. This mechanism also reduces the activation of calcitriol [189].

Worsening renal function reduces the conversion of 25(OH)D -25-hydroxyvitamin D- to 1,25(OH)_2_D (1,25-dihydroxyvitamin D) and limits vitamin D receptor (VDR) activation in the parathyroid gland, subsequently reducing negative feedback for PTH and ensuing gland hyperplasia and excess PTH production. The triad of low calcium, low calcitriol, and high phosphate triggers stimulation and causes parathyroid cell proliferation. This metabolic imbalance decreases the responsiveness of PTH to calcium and vitamin D, and worsening renal function prevents effective compensation, which leads to the appearance of bone mineral disorders (CKD-MBD) and vascular calcifications [190].

While PTH has been a long-established biomarker for measuring and monitoring the progression of secondary hyperparathyroidism, it has also been proposed as a biomarker for CKD [188]. The whole/intact PTH ratio (w/i PTH) has been demonstrated to correlate with eGFR in stage 3 of chronic kidney disease (CKD3), CKD4, and CKD5, as well as correlating with serum calcium in CKD5 [191]. The inflection point that was demonstrated in the correlation resides at 24.1 mL/min/1.73 m2 [191]. Studies further show that the w/i PTH ratio declines as CKD develops, and these changes in the ratio are associated with worsening kidney function, abnormal mineral metabolism, and renal outcome [191].

PTH has been recognized as an important independent risk factor for the increased incidence of CVD in CKD and overall mortality in this patient group [192]. Currently, second and third generation immuno-assays are the most commonly employed methods to measure PTH in clinical practice [192]. Seiler-Mussler et al. clearly demonstrated that second-generation PTH assays were more closely related to CV events, CKD progression, and overall all-cause mortality than non-oxidized PTH measurements in CKD patients not on dialysis [193].

Fibroblast Growth Factor 23 is a growth factor that is heavily involved in phosphate synthesis and vitamin D metabolism. Fibroblast Growth Factor 23 is a phosphotropic hormone that is predominantly secreted by osteocytes and, to a lesser degree, by mature osteoblasts [194]. It is part of the FGF family, which consists of two main subtypes: intracellular and extracellular FGF. Extracellular FGF is subdivided into endocrine and canonical, otherwise called paracrine [190]. These FGFs depend on heparin or heparan sulfate to act as a cofactor; however, endocrine FGFs 15/19/21 and FGF23 have a low affinity for heparin and heparan sulfate and so require Klotho proteins to act as coreceptors [195].

FGF23 itself is composed of three exons and two introns, which codify a 32 kDa glycoprotein composed of 251 amino acids [196]. The FGF23 gene is located on chromosome 12p3.3 and plays a major role as a crucial regulator of phosphate homeostasis through its effects on various organs.

FGF-23 is primarily secreted as a response to increased phosphate levels, where it proceeds to bind to FGFR1–Klotho complexes in the kidneys, resulting in the downregulation of the sodium phosphate co-transporters NaPi-IIa and NaPi-IIc [194]. Thus, it elevates the excretion of renal phosphate, thereby lowering serum phosphate levels.

It also acts as an inhibitor of renal 1-alpha-hydroxylase (CYP27B1) [197]. This limits the conversion of 25(OH)D to active 1,25(OH)_2_D (calcitriol) [195]. There is a continued upregulation of 24-hydroxylase, causing an enhanced degradation of the already synthesized 1,25(OH)_2_D (11), consequently reducing intestinal calcium and phosphate absorption and acting as an indirect factor of hypocalcemia [198]. The aforementioned FGFR–Klotho signaling also acts on the parathyroid glands inhibiting PTH [199]. Yet, in chronic kidney disease, there is a development of resistance to FGF-23 signaling, so PTH is persistently elevated, contributing to secondary hyperparathyroidism (SHPT).

Recently, other effects of FGFs-23 have been more intensely monitored. It has been shown that, outside of phosphate regulation, it also stimulates lung inflammation, is associated with elevated interleukin-6 and CRP in the liver [200] and with bone mineralization, increases synaptic density, and alters the morphology of hippocampal cells [195].

In CKD, FGF23 is of significant interest as a biomarker due to its elevation early in the course of the disease. Higher FGF 23 is associated with CKD progression in patients with established chronic kidney disease [197]. Yet, Stubbs et al. (2011) showed that significant increases in FGF23 gene expression in the bone do not occur until later CKD stages [201]. It is important to note that FGF23 levels in CKD rise [198] when calcium, phosphate, and PTH are not yet significantly changed [202].

While definite conclusions as to its usefulness in the clinical field would require dedicated clinical trials, there is significant potential for this biomarker, as it has already been demonstrated that high FGF23 is associated with an increased risk of new-onset CKD and higher all-cause mortality afterwards [203]. Furthermore, studies also show a close correlation between elevated FGF23 levels and a higher incidence of infection, as well as hospitalization, myocardial infarction, heart failure, and death in the CKD patient population [190].

Another significantly promising biomarker is sclerostin. Like FGF-23, it is also produced by osteocytes and has been associated with chronic kidney disease–mineral bone disorder (CKD-MBD) [204].

Sclerostin, a 22 kDa glycoprotein and a product of the SOST gene, is a soluble antagonist of the classical Wnt-beta signaling pathway [205] and regulates bone mass via Wnt-chain signaling. Like FGF-23, sclerostin has been found to be positively correlated with stages of CKD, beginning at CKD stage 2, and its levels increase with disease progression. Both FGF-23 and Sclerostin have been associated with bone turnover parameters [204]. However, unlike FGF-23, sclerostin’s presence is greater in cortical bone than in cancellous bone.

Overall, sclerostin has been linked with fewer osteoblasts and osteoclasts, and its elevation results in a thinner trabecular bone and a lower osteoid surface because of its correlation with decreased bone building, demonstrated through a lower activation frequency and bone formation rate, which occurs at high sclerostin levels [204]. Ji et al. (2018) have shown that sclerostin is elevated in serum in CKD3-CKD5 and is positively correlated with serum creatinine, blood phosphate [206], PTH, vitamin D, alkaline phosphatase, and calcium–phosphate products [207]. Sclerostin has been shown to be related to the calcium and phosphate mechanism, but there is still a lack of specific research regarding the exact mechanism of the correlation between calcium and phosphate in CKD-MBD. On the other hand, it has been negatively correlated with eGFR and blood calcium [207].

Serum sclerostin has been predicted as a potential sensitive indicator of the occurrence of CKD-MBD, and due to its close relationship to the prognosis of CKD, there has been a proposed association of sclerostin’s involvement in CKD pathogenesis [205]. Moreover, sclerostin has been shown to be related to metabolic disturbances, and a recent study demonstrated its relationship to hyperglycemia in non-dialysis CKD males specifically [206]. In pre-dialysis end-stage kidney failure (ESKD), serum sclerostin has been shown to have a positive correlation with pulmonary hypertension [208].

Dick-kopf-1 is a secreted antagonist of the Wnt pathway and inhibits the formation of the Wnt-Fzd-LRP5/6 trimeric complex [209]. It has an established role in embryonic development and bone formation in adults, where it is increasingly recognized as a major modulator of the metabolism of bone [210]. Elevated levels of DKK1 have also been observed in numerous human cancers.

However, more recently, it has emerged as a potential therapeutic target for CKD progression and the development of complications. DKK1 has been independently associated with low bone turnover, and in stages 3 and 4 of CKD, circulating levels of DKK1 and sclerostin have appeared to be predictive of bone disease [200]; however, further research is required to assess its efficacy as a potential biomarker in the diagnosis, treatment, and monitoring of renal osteodystrophy [209]. Serum levels of DKK1 were positively associated with faster progression to end-stage renal disease [209]. Several studies have reported lower levels of DKK1 in CKD as compared to controls, with levels dropping even early in the course of CKD [210], with the lowest being reported in CKD-4 [209]. Yet, Fang et al. (2014) demonstrated that increased renal production of DKK1 and circulating DKK1 levels were positively associated, and they hypothesized their relationship to CKD pathogenesis [211]. Further studies to gain a more precise insight into this mechanism and its relationship to CKD-MBD are required to validate it as a potential biomarker.

The biomarkers mentioned in the text are presented in Table 4.

## 7. Conclusions

There is a multitude of biomarkers related to CKD, with new ones emerging every year. The identification and implementation of novel biomarkers in CKD in clinical practice can result in significant advancements in early diagnosis, risk stratification, and personalized treatment strategies. These arising biomarkers may offer superior sensitivity in the detection of early kidney dysfunction and provide deeper insights into disease progression, correlated comorbidities, and potential therapeutic targets. However, the widespread clinical integration of these biomarkers faces challenges, mainly related to high costs and the necessity for standardized assays. The growing relevance of biomarkers in assessing kidney disease progression has been increasingly emphasized in the recent literature. Several reviews have explored advances in biomarker research for CKD [212,213,214,215], underscoring their potential in the comprehensive management of the disease, while also addressing the substantial challenges associated with their clinical implementation. It is essential to note that, whilst specific biomarkers are linked to differing CKD mechanisms and comorbidities, it is vital to recognize that many of the aforementioned biomarkers exhibit associations with multiple complications of CKD. This intersection is a reflection of the inherent complexity and multi-morbid nature that is characteristic of CKD. Due to this, the proposed division of biomarkers cannot be regarded as either definitive or exclusive. Acknowledging this inter-related nature of CKD is crucial for the appropriate interpretation of current and new biomarker data and for the successful development of comprehensive management strategies. The extension and continuation of research is pivotal for advancements in the diagnosis and treatment of CKD, and hopefully, as a result, a wider array of biomarkers will become accessible for use in daily clinical practice, enhancing the quality of healthcare delivered and improving patient outcomes for CKD.

## Figures and Tables

**Table 1:** Biomarkers of tubular secretion.

Biomarker	Origin/Source	Mechanism/Function	Clinical Relevance
KIM-1 (Kidney Injury Molecule-1)	Proximal tubule epithelial cells	Marker of proximal tubule injury	Elevated in AKI and CKD; correlates with ACR, associated with inflammation and fibrosis
NAG (N-acetyl-â-D-glucosaminidase)	Lysosomes of proximal tubule cells	Enzyme indicating tubular injury	Early marker of diabetic nephropathy (before albuminuria); reflects renal impairment and glycemic control
Uromodulin (Tamm–Horsfall Protein)	Thick ascending limb of Henle’s loop and distal convoluted tubule	Involved in ion transport, immunity, and prevention of kidney stones	Urinary and serum levels predict CKD onset and progression; reflects tubular damage and nephron mass
Klotho	Proximal and distal renal tubules; other tissues	Anti-aging and renoprotective molecule	Positive correlation with eGFR; deficiency promotes CKD progression
Dickkopf-3 (DKK-3)	Tubular epithelial cells in kidneys (expressed in response to injury)	Urinary stress glycoprotein marker; reflects ongoing renal tubular stress and injury	Elevated urinary DKK-3 linked to eGFR decline and CKD progression; associated with proteinuria and poor renal outcomes; predictor of CKD progression in COPD and resistant hypertension

Abbreviations: AKI, acute kidney injury; ACR, albumin–creatinine ratio; CKD, chronic kidney disease; COPD, chronic obstructive pulmonary disease; eGFR, estimated glomerular filtration rate.

**Table 2:** Inflammatory biomarkers.

Biomarker	Origin/Source	Mechanism/Function	Clinical Relevance
Ficolins	Liver, immune cells	Pattern recognition molecules; activate lectin pathway of the complement system	Elevated levels are associated with diabetic kidney disease progression and diabetes-related mortality; affect post-transplant graft outcomes
NLRP3 inflammasome	Immune system (intracellular complex)	Activates IL-1B and IL-18; stimulated by ROS and mitochondrial damage	Promotes inflammation, fibrosis, and CKD progression; considered a therapeutic target in hyperoxaluria, nephrocalcinosis, and renal fibrosis
TNFR1 and TNFR2 (tumor necrosis factor receptor 1 and 2)	Circulating receptors of TNF-alpha	Mediate inflammatory signaling; correlate with renal function	Early CKD biomarkers; predict progression in IgA nephropathy and DKD; prognostic in hypertension-related CKD
Calprotectin	Myeloid cells (especially neutrophils)	Acute-phase protein; released during neutrophil degranulation	Associated with new-onset CKD, vascular calcification, and increased cardiovascular and all-cause mortality in CKD patients
SIRI (systemic inflammation response index)	Derived from blood cell counts	Systemic inflammation index (Neutrophil × Monocyte/Lymphocyte)	Elevated in hypertensive patients at risk of CKD; predicts cardiovascular and all-cause mortality—especially useful in early CKD stages (I–III)
NLR (neutrophil-to-lymphocyte ratio)	Derived from blood cell counts	Marker of immune imbalance (Neutrophil/Lymphocyte)	High NLR correlates with advanced CKD, proteinuria, and decreased eGFR; predictive of cardiovascular risk and mortality in CKD and dialysis patients
CAR (CRP/albumin ratio)	Serum protein levels	Reflects systemic inflammation and nutritional status (PEW marker)	Reliable, accessible PEW and risk stratification marker

Abbreviations: Abbreviations: CKD, chronic kidney disease; CV, cardiovascular; eGFR, estimated glomerular filtration rate; DKD, diabetic kidney disease; NLR, neutrophil-to-lymphocyte ratio IL; interleukin, PEW, protein-energy wasting

**Table 3:** Cardiovascular biomarkers.

Biomarker	Origin/Source	Mechanism/Function	Clinical Relevance
Galectin-3	Macrophages, epithelial cells, fibroblasts	â-galactoside-binding lectin; mediates inflammation, fibrosis, cardiac remodeling	Elevated in CKD and heart failure. Predicts renal fibrosis, rapid renal function decline, and CV mortality. Correlates with serum creatinine and proteinuria. Potential early biomarker of both renal and cardiac complications.
sST2 (soluble suppression of tumorigenicity-2)	Cardiomyocytes, endothelial cells	Soluble form of ST2 receptor; binds IL-33, modulating inflammatory responses to cardiac stress	Predicts CKD progression and cardiovascular mortality. Especially valuable in dialysis patients.
GDF-15 (Growth differentiation factor-15)	Cardiomyocytes, kidneys, immune cells	Stress-induced cytokine involved in inflammation and oxidative stress	Associated with heart failure, atherosclerosis, and CKD progression. Predicts adverse renal and cardiovascular outcomes.
suPAR (soluble urokinase-type plasminogen activator receptor)	Immune system (monocytes, neutrophils), endothelium	Activates integrins, disrupting podocyte structure and filtration barrier	High levels linked with systemic inflammation, kidney function decline, and cardiovascular events. Predictive of mortality and CV risk in CKD.

Abbreviations: CKD, chronic kidney disease; CV, cardiovascular; IL, interleukin.

**Table 4:** Bone and mineral metabolism biomarkers.

Biomarker	Origin / Source	Mechanism / Function	Clinical Relevance
**Parathyroid Hormone (PTH)**	Parathyroid glands	Regulates calcium homeostasis	Established marker of SHPT and bone mineral disorder (CKD-MBD). A predictor of CKD progression, cardiovascular events, and mortality.
**Fibroblast Growth Factor 23 (FGF-23)**	Osteocytes and mature osteoblasts	Regulates phosphate metabolism. Suppresses 1-á-hydroxylase activity, reducing calcitriol production. Acts on parathyroid glands to suppress PTH.	Elevated early in CKD, before changes in phosphate, calcium, or PTH. Predicts CKD progression, all-cause mortality, and CV events.
**Sclerostin**	Osteocytes (mainly in cortical bone)	Reduces osteoblast activity and bone formation rate. Affects calcium–phosphate homeostasis.	Negatively correlated with eGFR. Promising marker for CKD-MBD and disease progression. Linked with hyperglycemia and pulmonary hypertension in CKD patients.
**Dickkopf-1 (DKK-1)**	Various tissues including bone; secreted protein	Regulates bone metabolism and embryonic development. Proposed involvement in “cross-talk” between tissues.	Emerging marker for CKD-related bone disorders and CKD-MBD. Levels correlate with disease severity and lower levels reported in advanced CKD. Associated with low bone turnover. Predictive of progression to ESKD.

Abbreviations: CKD, chronic kidney disease; CKD-MBD, chronic kidney disease–mineral bone disorder; CV, cardiovascular; ESKD, end-stage kidney disease, PTH, parathyroid hormone; SHPT, secondary hyperparathyroidism.
